# Effect of a Social Norm Email Feedback Program on the Unnecessary Prescription of Nimodipine in Ambulatory Care of Older Adults: A Randomized Clinical Trial

**DOI:** 10.1001/jamanetworkopen.2020.27082

**Published:** 2020-12-11

**Authors:** Fernando Torrente, Julián Bustin, Fabian Triskier, Nicolás Ajzenman, Ailin Tomio, Ricardo Mastai, Florencia Lopez Boo

**Affiliations:** 1 Institute of Neuroscience and Public Policy, INECO Foundation, Buenos Aires, Argentina; 2 Institute of Cognitive and Translational Neuroscience, CONICET, Ineco Foundation, Favaloro University, Buenos Aires, Argentina; 3 Instituto Nacional de Servicios Sociales para Jubilados y Pensionados, Buenos Aires, Argentina; 4 Sao Paulo School of Economics, São Paulo, Brazil; 5 Liver Transplant Unit, Hospital Alemán, Buenos Aires, Argentina; 6 Inter-American Development Bank, Washington, District of Columbia

## Abstract

**Importance:**

Nimodipine is a highly prescribed drug for the treatment of cognitive impairment and dementia in Argentina. There is little evidence to support the use of nimodipine for cognitive impairment and dementia.

**Objective:**

To test the effectiveness of a behavioral intervention based on social norm feedback to reduce prescription of nimodipine for cognitive impairment in Argentina.

**Design, Setting, and Participants:**

This pragmatic parallel-group randomized clinical trial included 2 arms with a 1:1 allocation ratio. General practitioner physicians in the national health care system for older adults in Argentina (INSSJP-PAMI) with history of high nimodipine prescription rate were enrolled. The study was conducted from May 2019 to October 2019, and data were analyzed from November 2019 to February 2020.

**Interventions:**

The treatment group received 2 emails with evidence-based information about nimodipine plus the individual’s level of nimodipine prescription compared with their peers. The control group received 2 emails with general information about the risks of overprescription in older adults.

**Main Outcomes and Measures:**

The primary outcome was the cumulative number of nimodipine prescriptions per 1000 prescriptions of all drugs made by the targeted physicians during the 6 months of the study. Secondary outcomes included annual monetary savings attributable to the intervention and physicians’ qualitative perceptions of the acceptability of the procedure.

**Results:**

Of 1811 physicians enrolled, 906 physicians (354 [39.1%] women; mean [SD] age, 57.10 [10.73] years) were randomized to treatment and 905 participants (331 [36.6%] women; mean [SD] age, 56.49 [10.47] years) to the control group. Physicians in the treatment group wrote a mean of 93.25 (95% CI, 89.27 to 97.24) prescriptions of nimodipine, compared with 98.99 (95% CI, 95.00 to 102.98) prescriptions among practitioners in the control group during the half-year of the intervention (mean difference, –5.73 [95% CI, –11.38 to –0.10] prescriptions; *P* = .046), which meant a 5.79% reduction. Regression analysis revealed a significant association of the group condition with number of prescriptions per 1000 total prescriptions when controlling for baseline prescriptions (B = –0.312 [95% CI, –0.465 to –0.160]; *P* < .001). The observed difference corresponds to a 4.48% reduction in nimodipine prescriptions per 1000 prescriptions of all drugs made by physicians in the treated group compared with the control group. Physicians who effectively opened the email in the treatment group (427 physicians [47.1%]) prescribed the drug 11.3% less compared with the control group (426 physicians) (mean difference, –10.78 [95% CI, –18.53 to –3.03] prescriptions; *P* = .006). Expenditures were 7.18% lower in the treatment group, resulting in an estimated annual net cost benefit of US $234 893.35 (95% CI, $225 565.35 to $237 112.30).

**Conclusions and Relevance:**

In this randomized clinical trial, the social norm email feedback program showed an effect on curbing the nonrecommended prescription of nimodipine. It was highly cost-effective and well accepted by participants.

**Trial Registration:**

ISRCTN.org identifier: ISRCTN17823729

## Key Points

**Question:**

Can a social norm–based behavioral intervention delivered by email reduce non–evidence-based prescriptions of nimodipine for treating cognitive impairment in older adults?

**Findings:**

This randomized clinical trial included 1811 high–nimodipine-prescribing physicians within the national health care system for older adults in Argentina. Physicians randomized to the social norm email treatment group reduced nimodipine prescribing after receiving 2 emails with evidence-based information about the drug and a comparison of the recipient ’s and peers’ rates of prescription compared with control physicians who received only information about the risks of overprescribing.

**Meaning:**

These findings suggest that reduction of non–evidence-based prescribing could be achieved through a highly cost-effective, noncoercive, and easily replicable behavioral intervention.

## Teaser

This randomized clinical trial examines the effect of a behavioral intervention email based on social norms feedback for physicians to reduce non–evidence-based prescriptions of nimodipine for cognitive impairment among older adults in Argentina.

## Introduction

The practice of prescribing drugs without evidence to support their use may become a significant public health problem by contributing to inappropriate polypharmacy in elderly patients and by increasing adverse drug effects and health system costs without reciprocal patient benefits.^1,2,3,4^ Elderly patients are often prescribed inappropriate medications and supplements for mild cognitive impairment and dementia.^5,6,7^ In Argentina, nonrecommended drugs account for between 41% and 45% of prescriptions for cognitive impairment, the most widely used of which is nimodipine^8,9,10^—a drug recommended for use with acute subarachnoid hemorrhage during short hospital stays, not outpatient use for cognitive impairment.^11,12,13^

Various strategies to change physicians’ prescribing practices have been attempted, ranging from continuing education, which is difficult and costly to scale up, to providing physicians with feedback on their prescribing behavior, which has provoked small but significant changes.^14^ Low-cost, innovative behavioral interventions known as *nudges* can also reduce inappropriate prescribing. Several studies have used social norm comparison, which intends to modify prescribing behavior by telling physicians how their prescribing practices compare with their peers.^15,16,17^ Such social norm comparisons have been successful in modifying specific behaviors in diverse public policy areas.^18^

Based on this principle, the main aim of this randomized clinical trial was to test a social norm feedback intervention’s effect in reducing unnecessary nimodipine prescriptions by general practitioners (hereafter, *physicians*) within the National Institute of Social Service for Retired and Pensioners (INSSJP-PAMI). This public institution provides free medical care to older people in Argentina and is the country and continent’s largest health insurance agency.

Our study also addresses 4 additional research considerations. First, email interventions have been largely unexplored, with contradictory indications about their effectiveness.^16,18,19^ However, hard-copy mailings can be difficult to conduct and costly to scale up,^20^ while email interventions are inexpensive and easily administered nationwide, which is especially important in developing countries with stringent operational and budget constraints. Second, to our knowledge, there have been no large-scale randomized clinical trials of behavioral interventions to improve prescribing practices in Latin America. Although social norm feedback in health care has been tried elsewhere, this intervention is in a new context.^21,22^ Third, given concerns about possible deterrence effects of communications with strong language or implying potential punitive actions from regulatory agencies,^20^ our intervention separates social norm and potential deterrence effects. Fourth, we assessed participants’ perceptions of the intervention in a postintervention survey.

## Methods

This randomized clinical trial was approved by the ethics committee of Fundación INECO and the Department of Legal Affairs of the INSSJP-PAMI. In agreement with national regulations^23^ and international guidelines,^24^ the committee waived physicians’ informed consent, as in similar studies,^15,17^ considering that there was minimal risk for participants, the results were of important social value, the research would not be practicable if consent was required, and participants may change their behavior if they were aware of taking part in research.^25^ The study did not involve patient contact, direct intervention, or access to patients’ personal information, as all information came from the INSSJP-PAMI electronic prescription register. Participating physicians did not complete additional measures or questionnaires for this study, except for an anonymous, voluntary survey that was sent after the intervention to the treatment group (Trial Protocol in Supplement 1). This study followed the Consolidated Standards of Reporting Trials (CONSORT) reporting guideline for randomized clinical trials.

### Study Design and Participants

We performed a pragmatic parallel-group randomized clinical trial of peer-comparison feedback emails to physicians with high nimodipine prescription rates to reduce excessive prescribing. The population comprised INSSJP-PAMI physicians who prescribed at least 1 nimodipine package during the last quarter of 2018, and the sample comprised the top 25% of nimodipine prescribers from that population. We removed extreme outliers (≥97.5th percentile) from this sample to prevent distorting effects on statistics due to measurement error or exceptionally deviant behavior from the norm.

### Randomization

A study investigator (A.T.) analyzed the 2018 database of nimodipine prescriptions for selecting physicians who met inclusion criteria. Selected participants were randomly allocated to control and treatment groups with a 1:1 ratio. A simple randomization procedure was performed with R statistical software version 3.5.3 (R Project for Statistical Computing). After randomization, we tested for differences between groups in the number of nimodipine prescriptions over the last trimester of 2018, and we found no differences. The investigator involved in selection did not participate in the implementation of the interventions nor in the statistical analysis of the results.

### Intervention

The email intervention consisted of transmitting good-practice prescribing information to physicians with high nimodipine prescription rates. Both the control and treatment group messages were framed as part of a communications campaign for improving pharmacological practice quality. During the intervention, all physicians received 2 emails. The treatment group’s first email included 2 components: evidence-based information about adequate nimodipine use and a comparison of the individual’s and peers’ nimodipine prescriptions (eAppendix 1 in Supplement 2). The second email, delivered 3 months later, included information on changes in the physician’s nimodipine prescriptions during the previous quarter (ie, change or no change). Emails for physicians who reached a relative reduction of 10% of prescriptions compared with their baseline mean acknowledged this success as verbal reinforcement of the positive behavior (called the *acknowledgment version*) (eAppendix 3 in Supplement 2). The email sent to those who did not reach the 10% target was intended to potentiate the social norm component by stating that many of their colleagues had reduced nimodipine prescribing after the first email and encouraging a change (called the *encouragement version*) (eAppendix 4 in Supplement 2). Both messages repeated the evidence-based information about nimodipine. For the control group, the first message contained general information about the inconveniences of unnecessary drug prescription and polypharmacy in older adults and links to medical guidelines to improve prescription practices (eAppendix 2 in Supplement 2). Three months later, the control group was sent a second email with information about the risks and complications from benzodiazepines use in older adults (eAppendix 5 in Supplement 2).

Every feedback message was carefully worded to avoid any pressure from regulatory authorities or aversive real or imagined consequence of the intervention, as we sought to control for deterrence effects and preserve physicians’ freedom to choose. To isolate social norm and potential deterrence effects, we used terms, such as *invite* or *consider*, to emphasize the physician’s decision-making power and omitted mention of duties, regulations, or penalties. Every email was signed by the high-profile medical director of INSSJP-PAMI to try to raise the messages’ credibility, rather than a regulatory or auditing authority who could have caused intimidation, fear, or automatic obedience instead of the desired behavioral effects.

### Outcome

The primary outcome was measured at the physician level and defined as the cumulative total number of nimodipine prescriptions per 1000 prescriptions of all drugs by the targeted physicians for 6 months after the first email was sent out (May to October 2019). Each physician’s total was registered monthly for 1 year prior to the first email (baseline period divided in two 6-month periods, hereafter *baseline period 1* and *baseline period 2*) and for 6 months afterward (ie, intervention period). One nimodipine prescription equals 1 dispensed nimodipine package as registered in the electronic filing system. The total numbers of each physician’s prescriptions for all other drugs were also registered, but we were not able to discriminate the types of drugs prescribed besides nimodipine. Our relative prescription measure alleviates potential measurement problems from physicians’ holidays and other unknown sources of variability. The primary outcome was analyzed on an intention-to-treat basis.

In addition to the main analysis, we estimated the effect of the intervention on direct prescription costs, using the retail price at the time of the dispensation registered by the INSSJP-PAMI system. We also performed a subsample analysis of physicians who opened the emails to measure the potential effect of our intervention on those treated. Finally, we qualitatively assessed intervention acceptability for treatment group participants.

### Statistical Analysis

A multiple linear regression analysis was applied to evaluate the association of the primary outcome with the group condition, controlling for baseline prescription levels. To test the intervention’s effects over time, we performed a 3 (time: baseline 1, baseline 2, intervention) × 2 (group: treatment, control) repeated measures analysis of variance (ANOVA) with nimodipine prescriptions per 1000 total prescriptions as the dependent variable.

One-way ANOVA comparisons were performed to test between-group differences in pooled prescriptions during baseline and interventions periods and to test month-by-month differences inside the intervention period. Finally, the Pearson χ^2^ test was used to analyze association between categorical variables such as treatment condition, sex, and opening rate of the emails. Power calculation with GPower 3.1 indicated that a sample of 1030 participants would be sufficient to detect an effect size of 0.5% in the lower bound of those reported in the feedback interventions literature,^14,15,26^ with 80% of statistical power and a significance level of .05 in the repeated measures ANOVA. Considering the negligible costs of the intervention, very small changes would be still affordable. *P* values were 2-sided. Data were analyzed from November 2019 to February 2020.

## Results

**Figure 1.**
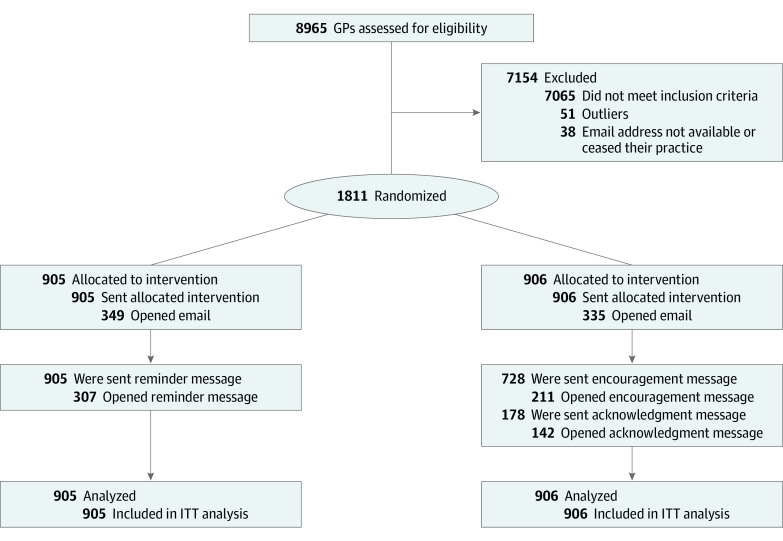
Flowchart of Study Recruitment

Of 8965 eligible general practitioner physicians, 7430 prescribed nimodipine at least once during the last quarter of 2018, and 1900 were at or above the 75th percentile of nimodipine prescribers established as inclusion criteria. From this group, 51 physicians were removed as outliers and 38 physicians were excluded because their personal email addresses were not available or because they ceased working for the INSSJP-PAMI before randomization (Figure 1). Of the final sample of 1811 physicians, 906 (354 [39.1%] women; mean [SD] age, 57.10 [10.73] years) were randomized to treatment, and 905 (331 [36.6%] women; mean [SD] age, 56.49 [10.47] years) were randomized to the control group (Table 1). All were included in the intention-to-treat analysis. The treatment and control versions of the first email were sent to all participants in each group. According to the mailing statistics, 335 emails (37.0%) were opened for the treatment group, and 349 emails (38.6%) were opened for the control group (eTable 1 in Supplement 2). The 2 study conditions showed no significant differences in opening rate of the emails (χ^2^ = 0.486; *P* = .49). This parity helps rule out observed differences as differences in reaching the groups. Three months after the first email, the second round of emails were sent: the acknowledgment version email was sent to 178 treatment group physicians who reached the 10% reduction target during the intervention’s first trimester. The rest of the treatment group was sent the encouragement version email. The control group received a message about benzodiazepines use as its second email. The acknowledgment email was opened by 142 recipients (79.8%), while the encouragement email was opened by 211 recipients (29.0%), and the control email by 307 recipients (33.9%); the rate of opening was significantly different (χ^2^ = 164.27; *P* < .001) (eTable 2 in Supplement 2). Among physicians in the treatment group who did not open the first email, 92 (10.2%) opened the second email, so that 427 physicians (47.1%) read either the first or the second email with the social norm feedback message (eTable 3 in Supplement 2). There were differences in age, years of practice at the institute, and sex in opening rates of the emails (eAppendix 6 in Supplement 2).

**Table 1.** Characteristics of Study Participants at Baseline

Characteristic	Control (n = 905)	Treatment (n = 906)
Age, mean (SD) y.	56.49 (10.47)	57.10 (10.73)
Women, No. (%)	331 (36.6)	354 (39.1)
Years of practice, mean (SD)^a^	8.49 (3.09)	8.47 (3.07)
Nimodipine prescriptions, No.		
Baseline period 1^b^	94 699	91 905
Baseline period 2^c^	93 051	91 880

^a^ Includes only time in practice at the Instituto Nacional de Servicios Sociales para Jubilados y Pensionados.

^b^ May to October 2018.

^c^ November 2018 to April 2019.

### Primary Outcome Comparisons

During the intervention period, the treatment group prescribed 5.7% less nimodipine units compared with the control group (84 489 units vs 89 588 units) (Table 2). Treated physicians made a mean of 93.25 (95% CI, 89.27 to 97.24) prescriptions of nimodipine compared with 98.99 (95% CI, 95 to 102.98) prescriptions among the control group during the intervention period (mean difference, –5.73 [95% CI, –11.38 to –0.10] prescriptions; *P* = .046). The mean number of nimodipine prescriptions per 1000 total prescriptions of any drugs (primary outcome) during the intervention period was 7.00 (95% CI, 6.74 to 7.26) in the control group and 6.6 (95% CI, 6.34 to 6.85) in the treatment group, a relative difference of 5.71% (Table 3). A regression analysis revealed a significant association of the group condition with the primary outcome variable when controlling for baseline prescriptions (B = −0.312 [95% CI, –0.465 to –0.160]; *P* < .001; *R*^2^ = 0.828). The observed difference corresponds to a 4.48% reduction in nimodipine prescriptions per 1000 prescriptions of all drugs in the treated group compared with the control group.

**Table 2.** Nimodipine Prescriptions During Intervention Period

Group	Total prescriptions			Prescriptions per prescriber			
	Control, No.	Treatment, No.	Difference, No. (%)	Mean (95% CI)			*P* value
				Control	Treatment	Difference	
Total sample^a^	89 588	84 489	–5099 (–5.69)	98.99 (95.00 to 102.98)	93.25 (89.27 to 97.24)	–5.73 (–11.38 to –0.10)	.046
Partial sample^b^	40 625	36 116	–4509 (–11.10)	95.36 (89.88 to 100.85)	84.58 (79.10 to 90.06)	–10.78 (–18.53 to –3.03)	.006

^a^ Includes 906 physicians randomized to the treatment group and 905 physicians randomized to control group and included in intention-to-treat analysis.

^b^ Includes 427 physicians in the treatment group and 426 physicians in the control group who opened the first or second email.

**Table 3.** Effect of the Intervention on Number of Nimodipine Prescriptions per 1000 Total Drugs Prescriptions

Prescriptions	Prescriptions per 1000 total prescriptions, mean (95% CI)		*P* value
	Control (n = 905)	Treatment (n = 906)	
Pooled			
Baseline period 1^a^	7.69 (7.44-7.95)	7.54 (7.28-7.81)	.42
Baseline period 2^b^	7.53 (7.26-7.79)	7.44 (7.17-7.71)	.66
Intervention period^c^	7.00 (6.74-7.26)	6.60 (6.34-6.85)	.03
Monthly			
May 2019	7.18 (6.86-7.50)	6.81 (6.51-7.12)	.11
June 2019	7.15 (6.85-7.45)	6.83 (6.53-7.13)	.15
July 2019	6.90 (6.61-7.19)	6.69 (6.40-6.99)	.33
August 2019	7.03 (6.73-7.33)	6.57 (6.29-6.86)	.03
September 2019	6.85 (6.55-7.15)	6.27 (5.96-6.57)	.01
October 2019	6.64 (6.33-6.95)	6.13 (5.84-6.42)	.02

^a^ May to October 2018.

^b^ November 2018 to April 2019.

^c^ May to October 2019.

**Figure 2.**
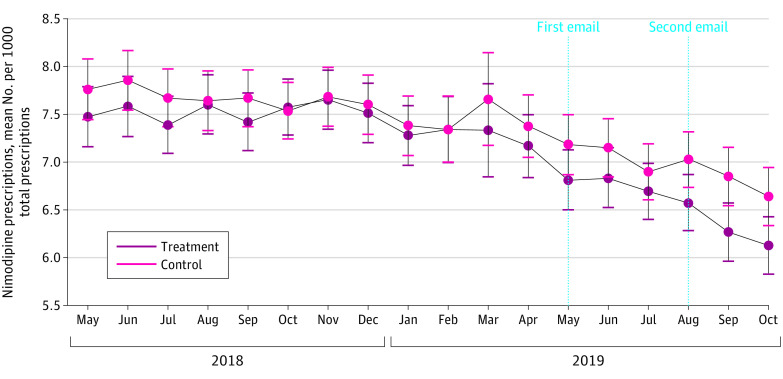
Effects of the Intervention on Primary Outcome

Multivariate testing showed a significant main effect of time (Pilai Trace = .162; F_2,1808_ = 174.85; *P* < .001; *d* = .88). and a Time × Group interaction (Pilai Trace = .008; F_2,1808_ = 7.60; *P* = .001; *d* = .18). Within-participant tests demonstrated a significant effect of time in the treatment (F_2,1810_ = 146.52; *P* < .001; *d* = .80) and control (F_2,1808_ = 94.12; *P* < .001; *d* = .64) groups. Between-group comparisons (Table 3) revealed that treatment group physicians wrote significantly fewer nimodipine prescriptions per 1000 total prescriptions during the pooled intervention period (ie, May-October 2019) than control physicians (mean difference, –0.403 [95% CI, –0.770 to –0.035]; *P* = .03.), but not during the baseline periods (Table 3). Additionally, a month-by-month analysis yielded significant between-group differences from August to October 2019, suggesting a cumulative effect or a beneficial impact of the second email (Table 3 and Figure 2).

### Subgroup Analysis

During the intervention period, physicians who had opened either of the first or second emails prescribed 11.1% less nimodipine units than control physicians who opened either of the first or second emails (36 116 prescriptions vs 40 625 prescriptions; mean difference per prescriber, –10.78 [95% CI, –18.53 to –3.03] prescriptions; *P* = .006). Regression analysis for this subsample revealed a significant association of the group condition with nimodipine prescriptions per 1000 total prescriptions during the intervention period when controlling for baseline prescriptions (B = –0.671 [95% CI, –0.900 to –0.442]; *P* < .001; *R*^2^ = 0.816).

### Cost-Effectiveness Analysis

The expenditures for the treatment group were 7.18% lower than those of the control group over the intervention term (Argentine pesos $55 035 885 vs $59 290 929 [US $691 497.28 vs $744 959.69]) (eTable 4 in Supplement 2). We estimate that if this intervention were implemented for all eligible physicians in the country for 1 year, the intervention could save US$234 893.35 (95% CI, 225 565.35 to 237 112.3) (Trial Protocol in Supplement 1).

### Postintervention Survey

Among 906 physicians in the intervention group, 102 physicians (11.3%) completed the postintervention survey. Of these, 96 physicians (94.1%) claimed to have read the emails. The messages were classified as useful or very useful for their medical practice by 76 respondents (74.5%), and 91 respondents (89.2%) acknowledged having modified their behavior because of the messages (a lot: 20 respondents [19.6%]; quite a lot 47 respondents [46.1%]; and moderately: 24 physicians [23.5%]). Regarding the importance of the messages’ components, 84 respondents (82.4%) stated that the information on scientific evidence was important or very important, 74 respondents (72.5%) said the feedback on their nimodipine prescription rates was important or very important, 61 respondents (59.8%) said that comparative information with colleagues (ie, social norm) was important or very important, and 96 respondents (94.1%) said that information about the risks of overprescription and polypharmacy was important or very important. Also, 94 respondents (92.2%) considered these messages to constitute an adequate or totally adequate procedure to improve the quality of the prescribing, while 8 respondents (7.8%) declared themselves neutral, and no respondents considered the intervention inappropriate. Finally, 95 respondents (93.1%) reported they would agree to receive new communications of this kind, 5 respondents (4.9%) were neutral, and 2 respondents (1.9%) declined.

## Discussion

The randomized clinical trial aimed to test the effectiveness of a behavioral intervention based on social norm feedback to reduce nonrecommended nimodipine prescriptions for preventing or treating cognitive impairment. It used an experimental randomized controlled design targeted to physicians on a national scale. Our results confirmed the procedure’s effectiveness in an intention-to-treat analysis, as the treatment group prescribed the drug 5.69% less than the control group during the 6 months of the intervention, and the physicians who opened the email prescribed 13% less nimodipine compared with the control group. Previous literature on social norms use in public policy reported effect sizes ranging approximately 10 percentage points, with a low end at 1 to 2 percentage points, middle at 2 to 5 percentage points, and high end at 5 to 10 percentage points.^18^ Thus, our results are in the medium and high levels of expected effects for this kind of intervention. The 5.69% prescription reduction in our study is higher than the 3.3% reported by Halsworth et al^15^ and lower than the 11.1% Sacarny et al^17^ found over a 9-month period. However, when considering only physicians who opened the emails, the effect is similar to that of Sacarny et al.^17^

The control group also showed a significant within-group reduction of prescriptions during the intervention phase. As a first hypothesis, we posited a natural decreasing trend in nimodipine use over time. However, the prescription rates were fairly stable during the 2 preceding 6-month periods. As a second hypothesis, we cannot discard the possibility that the control group was not completely blind to the messages directed to the treatment group. Communication between colleagues on prescription drugs is a usual, desirable practice of medical teams. Any contamination may have reduced the differences between the groups but not the intervention’s impact and may have unintentionally reduced prescriptions among the whole sample.

The intervention’s impact was also evident in several measures. It reduced expenditures and was inexpensive, so it may be considered highly cost-effective. Our study also supports the efficacy of social norm feedback as a behavioral tool for modifying physicians’ prescribing and expands the cultural diversity of existing studies. Furthermore, we were able to isolate deterrence effects from other components of the nudge without compromising the intervention’s effect. We tested the feasibility of sending emails instead of mail with positive results, since our effects were in the range of earlier studies using mailed letters. The lower costs, speed, and simplicity of emails may make them a more efficient option that has been sparsely used until now. However, the subgroup analysis of participants who reliably opened the emails demonstrates that there is much room for improvement regarding the intervention’s effectiveness. Because this group showed a more pronounced reduction in nimodipine prescriptions than the entire sample, it may suggest that if more physicians read the emails, the intervention’s effect would be even greater.

Future studies may want to evaluate how to improve the rate that emails are opened to augment the intervention’s potency. The email subject line could have considerable sway. Likewise, a possible next step is to examine how the population not included in the trial can be approached with different social norm variants. Our positive results encourage replication of the intervention for other nonrecommended drugs, although the escalation and replication of such procedures in the same population will require exploration of possible saturation effects.

Finally, most postintervention survey respondents reported that the messages influenced their prescribing behaviors. All components were considered important, but paradoxically, the social norm component was the least valued element—suggesting that perhaps people frequently misperceive what influences their behavior. Crucially, virtually all respondents considered the intervention appropriate, and most would agree to receive similar communications again. The survey results suggest that the intervention was generally well accepted.

### Limitations

This study has several limitations. First, the intervention was targeted to the top nimodipine prescribers, so we cannot know that other physicians would react the same way. Second, we cannot reject the possibility that spontaneous communications between the experimental groups contaminated the experiment. Third, despite focusing the nudge toward well-defined behavioral components and explicitly avoiding confounding factors, the 2-arm design did not allow us to evaluate specific behavioral effects inside the nudge. Fourth, without using emailed and mailed letters, we cannot estimate the impact a mailed letter would have had. Fifth, we could not know if the treated physicians changed their prescriptions from nimodipine to other nonrecommended drugs. Sixth, we cannot estimate at present the long-standing effects of the treatment with our time-limited data.

## Conclusions

This randomized clinical trial of a behavioral intervention based on social norm feedback and information delivered through email was effective in reducing nonrecommended prescriptions of nimodipine for cognitive impairment among physicians on a national scale. The experiment also shows that it is possible to modify physicians’ behavior without coercion while preserving their freedom to choose. Since the intervention was highly cost-effective, well-accepted, and easily replicable, we believe it has the potential to become a useful and efficient public policy tool in diverse cultural contexts.

## Notes

Supplement 1. **Trial Protocol**

Supplement 2. **eAppendix 1.** First Active Letter **eAppendix 2.** First Control Letter **eAppendix 3.** Second Active Letter: Acknowledgement Version **eAppendix 4.** Second Active Letter: Encouragement Version **eAppendix 5.** Second Control Letter **eTable 1.** Rate of Opening the First Email **eTable 2.** Rate of Opening the Second Email **eTable 3.** Global Rate of Opening the Emails **eTable 4.** Expenditures of Nimodipine Prescriptions **eAppendix 6.** Additional Statistics

Supplement 3. **Data Sharing Statement**
