# Supplementary material for: Effect of a Social Norm Email Feedback Program on the Unnecessary Prescription of Nimodipine in Ambulatory Care of Older Adults: A Randomized Clinical Trial
Source: JAMA Netw Open. 2020 Dec 11;3(12):e2027082. doi: 10.1001/jamanetworkopen.2020.27082 (PMC7733153; doi:10.1001/jamanetworkopen.2020.27082)
Supplement: Supplement 1. — Trial Protocol [file jamanetwopen-e2027082-s001.pdf]

Protocol:

Effect of social norm feedback e-mails on high-prescribers of Nimodipine in older adults: a randomized controlled trial

Fernando Torrente

Julián Bustin

Fabián Triskier

Nicolás Ajzenman

Ailin Tomio

Ricardo Mastai

Florencia Lopez Boo

Abstract

Background: The prescription of drugs without evidence is a significant public health problem. It can also result in large efficiency losses in the use of public resources. The aim of this study will be to test the effectiveness of a behavioral intervention based on social norm feedback to reduce non-recommended prescription of nimodipine as a drug for cognitive impairment in Argentina.

Methods: We will perform a pragmatic randomized trial of peer comparison feedback delivered through e-mails to high nimodipine-prescribing general practitioners (GPs) of the national health-care system for older adults (INSSJP-PAMI). Eligible GPs will be randomized to a treatment or to a control condition. The intervention group will receive two e-mails with evidence-based information about nimodipine plus the participant’s level of prescription of nimodipine compared to their peers. The control arm will receive also two e-mails with general information about the risks of over-prescription and about the risk of overuse of benzodiazepines in older adults. The primary outcome will be the cumulative number of nimodipine prescriptions per 1000 prescriptions of all drugs made by the targeted practitioners during 6 months after the intervention started.

Discussion: The results of this study could have several potential implications for improving the quality of medical practice and public policy in the health area. It would provide an efficient tool for aligning nonrecommended prescription with the evidence and for reducing unnecessary costs without employing coercive methods and preserving the practitioners’ freedom to choose. Besides, the trial would allow to test the cross-cultural validity of the social norms feedback procedure and the feasibility of implementing it via e-mail.

Trial registration: The trial is intended to be registered at the ISRCTN registry.

Keywords:

nimodipine – peer comparison – nudge – behavioral insights – older adults – medication overuse

Administrative information

Title {1}	Effect of social norm feedback e-mails on high-prescribers of Nimodipine in older adults: a randomized controlled trial
Trial registration {2a and 2b}.	The trial is intended to be registered at the ISRCTN registry.
Protocol version {3}	February 2019
Funding {4}	The study will be partially funded by the Interamerican Development Bank
Author details {5a}	Institute of Neuroscience and Public Policy, INECO Foundation (FT)<br>Institute of Cognitive and Traslational Neuroscience (CONICET- Ineco Foundation, Favaloro University) (FT, JB, FTR)<br>Instituto Nacional de Servicios Sociales para Jubilados y Pensionados (INSSJP-PAMI), Argentina (JB, FTR, RM)<br>Sao Paulo School of Economics (NA)<br>Interamerican Development Bank (FLB)
Name and contact information for the trial sponsor {5b}	INECO Foundation<br>Address. Pacheco de Melo 1854<br>City: Buenos Aires<br>Country. Argentina<br>Zip. C1126AAB<br>Tel. +54 (0)11 48120010<br>Contact: Dr. Teresa Torralva<br>Email: ttorralva@ineco.org.ar
Role of sponsor and funders {5c}	FLB, as a member of the funding organization, is also part of the research team that designed the study and will participate in the writing of the report and its publication, although she will not have ultimate authority over any of these activities.

Introduction

Background and rationale {6a}

The prescription of drugs without evidence is a significant public health problem. In the case of people over 65 years, polypharmacy and adverse effects could have negative consequences for health and quality of life. Also, undue prescriptions cause burdensome costs to the health system without any benefit for the patient. Among the medications improperly prescribed in the population of older adults, there is a diverse set of drugs used for the treatment of patients with mild cognitive impairment and dementias, including antioxidants, nootropic agents, vitamins, biological products and vasodilators (Rojas et al., 2010; Rojas et al., 2013). Currently 46.8 million people live with dementia across the world, and this number is expected to double every 20 years, reaching an estimated number of people living with dementia as high as 131.5 million by 2050 (Prince et al., 2015). The only worldwide recommended pharmacological treatment for different dementias are the acetylcholinesterase enzyme inhibitors (donepezil, galantamine and rivastigmine), and the NMDA receptor antagonist memantine (NICE, 2018; McKeith at al., 2017; Schmidt et al., 2015). In the case of Mild Cognitive Impairment, a pre-dementia syndrome, there is no recommended drug for its treatment (Petersen et al., 2018).

Previous reports in Argentina revealed that the prescription of recommended drugs for the treatment of cognitive impairment accounted for between 54 and 59% of total indications, meanwhile non-recommended drugs represented between 41 and 45 %. Among these, the most used drug was nimodipine (Rojas et al., 2010, 2013). According to the current evidence, nimodipine has a single indication for the treatment of acute subarachnoid hemorrhage, which is usually administered for short periods during the hospitalization period. There is not enough evidence for the use of nimodipine in the prevention or treatment of cognitive impairment on an outpatient and prolonged basis.

Earlier studies have shown behavioral interventions known as “nudges” can reduce inappropriate drug prescribing among physicians. Specifically, some studies employed social norm comparison, an approach that intends to modify prescribing behavior by comparing physicians’ prescriptive performance with that of their peers (Hallsworth et al., 2015; Meeker et al., 2016; Sacarny et al., 2018). The comparison against a social norm has an important influence on individual’s behavior and has been proved to be successful for modifying specific behaviors in diverse areas of public policy (John, Sanders, & Wang, 2014). Although social norm feedback has been tried in the health area as an effective tool in other world regions, no large-scale randomized controlled trials of behavioral interventions have been carried in Latin America for improving inadequate prescription

patterns by physicians (Sanders, Snijders, & Hallsworth, 2018).
Also, most previous studies employed physical paper letters to deliver the messages, the question of how this type of treatment affects behavior when delivered through e-mail has been largely unexplored. This question is especially relevant from a policy point of view, especially in developing countries, where operational and budget constraints could be stringent. Communications through physical mailing may be difficult to implement and replicate at large scale (Liao & Navathe, 2018). In contrast, e-mail interventions are inexpensive and easily scaled, hence they could be a useful alternative to modify prescriptive patterns of physicians at national scale. However, some authors suggest that e-mail communications may be less effective than paper letter communications (John, Sanders, & Wang, 2014).
Third, some concerns have been raised about the possible deterrence effects of feedback letters in previous studies that included strong language and that implied potential punitive actions from regulatory agencies (Liao & Navathe, 2018). The use of coercive wording or allusions to potential punishments from authorities may have obscured the mechanisms of the intervention turning difficult to separate the social norm effect from a potential deterrence effect.

Objectives {7}

The main aim of this study will be to test the effectiveness of a social norm feedback intervention to reduce the unnecessary prescription of nimodipine by general practitioners within the National Institute of Social Service for Retired and Pensioners (INSSJP-PAMI) in Argentina.
As secondary objectives, we will address four additional issues. First, we will assess the effectiveness of e-mails as delivering method of the behavioral intervention. Second, we will evaluate the validity of social norms feedback as a procedure for changing physicians prescriptive practice in the Latin American context. Third, given concerns about possible deterrence effects of communications with strong language or implying potential punitive actions from regulatory agencies, our intervention separates social norm and potential deterrence effects avoiding the use of coercive wording and obviating any mention to potential punishments from authorities. Fourth, we are also interested in knowing more about the acceptability of the intervention from the participants' point of view. For this purpose, a brief post-intervention survey will be implemented.
We hypothesize that the treated group will reduce their rates of nimodipine prescriptions in comparison with the control group. We also expect that the intervention will be cost-effective and well accepted by the participants.

Trial design {8}

Pragmatic parallel group randomized controlled trial with two arms (1:1 allocation ratio).

Methods: Participants, interventions and outcomes

Study setting {9}

The study will be entirely carried at the National Institute of Social Services for Retired and Pensioners (in Spanish, Instituto Nacional de Servicios Sociales para Jubilados y Pensionados, INSSJP). The INSSJP was created in 1971 with the objective of providing universal coverage of social and health services to the specific needs of the retired and pensioners of Argentina. The INSSJP is the largest health insurance agency in Argentina and South America, with 5.089.021 affiliates in 2015. Approximately, 65% of the Argentinean citizens older than 60 years old are affiliates of the INSSJP.

Eligibility criteria {10}

The initial study population will be the general practitioners of the INSSJP-PAMI system who prescribed at least one package of nimodipine during the last quarter of 2018 (n = 8965). The sample under study will be the high prescribers within that population, defined as the top 25% prescribers of nimodipine during the last quarter of 2018.

Who will take informed consent? {26a}

As in previous similar studies (Hallsworth et al., 2015; Sacarny et al., 2018) the present trial will not require informed consent from prescribers, since obtaining consent would invalidate the results. The study did not involve contact or direct intervention on patients, nor access to their personal information.

Additional consent provisions for collection and use of participant data and biological specimens {26b}

Not applicable

Interventions

Explanation for the choice of comparators {6b}

The control condition is conceived as a non-specific placebo comparator that replicates the format of the treatment intervention without its active ingredients (evidence-based information about nimodipine plus peer comparison). The comparator is supposed to control for non-specific behavioral factors such as receiving attention or raising awareness about over-prescription as a general problem in medical practice. Also, the placebo e-mails will allow to check if both groups open the e-mails at similar rates.

Intervention description {11a}

During the intervention phase of the study, participants in the two arms of the trial will receive two e-mail communications. The messages will be framed as a communicational campaign for improving the quality of pharmacological practice. In the case of the treatment arm, the first communication will include two components: a) evidence-based information about the adequate use of nimodipine and b) the participant's level of prescription of nimodipine compared to their peers. The second message, 3 months later, will include information on nimodipine prescriptions' change observed by the participant during the previous quarter ("change" or "no change"). The e-mail directed to those that reached a relative reduction of 10% of prescriptions compared to their baseline average will include an acknowledgement of their success intended to work as a verbal reinforcement of the positive behavior ("acknowledgement version"). The e-mail to the rest of participants will intend to potentiate the social norm component by stating that many of their colleagues reduced their prescriptions of nimodipine after the first e-mail and by encouraging them to revise their prescriptive practice ("encouragement version"). In the two modalities, the evidence-based information about nimodipine will be repeated. In the case of the control arm, the first message will contain general information about the inconveniences of unnecessary drug prescription and polypharmacy in older adults and links to medical guidelines to improve the prescription. Three months later, a second e-mail with information about the risks and complications of the use of benzodiazepines in older adults will be sent to the control group. Communications by e-mails will avoid any coercive or intimidating wording and they will purposively obviate any mention to potential punishments from authorities. Moreover, the expected change in prescriptive behavior will be framed as a free medical decision.

Criteria for discontinuing or modifying allocated interventions {11b}

Not applicable.

Strategies to improve adherence to interventions {11c}

The e-mailing system provides an estimation of how many participants open the e-mail, therefore we will consider this estimate as a proxy of the reach of the intervention. Accordingly, non-adherence could be understood as the omission of reading the e-mails. For our primary analysis, we decided to carry out an intention-to-treat approach integrating both the participants who opened the e-mail and those who did not. As a secondary subgroup analysis, we considered separately those participants that opened the e-mail. We have no plans for increasing the opening of the e-mails during the trial.

Relevant concomitant care permitted or prohibited during the trial {11d}

Not applicable

Provisions for post-trial care {30}

Not applicable

Outcomes {12}

The primary outcome will be the cumulative total number of nimodipine prescriptions per 1000 prescriptions of all drugs made by the targeted practitioners during the 6 months after the intervention started.

As secondary outcome, we will estimate the annual monetary savings attributable to our intervention at the national level (for details see Statistical Methods section). Also, we will perform a sub-sample analysis of the physicians that open the e-mails, as informed by the mailer system. Finally, we will assess the acceptability of the intervention through a brief *ad hoc* survey to the intervention arm participants at the end of the study (six months after the start of the intervention).

Participant timeline {13}

The number of nimodipine prescriptions for each physician is registered monthly during a year prior to the first e-mail (baseline period) and during the semester after (intervention period). Follow-up period will eventually comprise a year after the intervention period ends.

Sample size {14}

To calculate our minimum sample size, we used GPower 3.1. Power calculation estimated a sample size of 1030 participants for detecting an effect size of d=0.05, with 80% of statistical power and a significance level of 0.05 in the repeated measures ANOVA.

Recruitment {15}

As a first step, all the physicians that prescribed nimodipine during the last quarter of 2018 were selected. As a second step, we removed outlier prescribers. Based on previous literature (Hallsworth et al., 2015), the outlier threshold was set at the 97,5th percentile of nimodipine box prescriptions over the last trimester of 2018. As a third step, once outliers were removed, the top 25% prescribers of nimodipine were selected. From this sample, all the participants that remain as providers at the INSSJP-PAMI before the intervention begins and whose e-mail address is active will be randomized to control or treatment arm.

Assignment of interventions: allocation

Sequence generation {16a}

Selected prescribers will be randomly allocated to control and treatment (1:1 ratio) controlling for number of nimodipine prescriptions over the last trimester of 2018. Randomization is performed with R software with the package RandomizeR.

Concealment mechanism {16b}

Not applicable

Implementation {16c}

A study investigator (AT) analysed the 2018 database of prescription of nimodipine for selecting those who met inclusion criteria. Before the intervention begins, she will randomly assign the selected participants to treatment or control groups. AT will not participate in the implementation of the interventions nor in the statistical analysis of the results.

Assignment of interventions: Blinding

Who will be blinded {17a}

Participants in treatment group will likely be aware of the interventions they were assigned to but unaware that they were involved in a trial. Physicians names and other identificatory information is accessible only to the principal investigator (FT) for matching successive monthly waves of prescription rates, but this information will be blind to any other team group and INSSJP-PAMI authorities.

Procedure for unblinding if needed {17b}

Not needed.

Data collection and management

Plans for assessment and collection of outcomes {18a}

All data is obtained from the prescriptions database of the INSSJP-PAMI.

Plans to promote participant retention and complete follow-up {18b}

Since the intervention does not involve any evaluation interview or the completion of questionnaires by the participants, there is no mechanism in place to ensure retention. All the necessary data is obtained from the INSSJP-PAMI prescription system that automatically records the prescribing behavior of physicians.

Data management {19}

All data is obtained from the prescriptions database of the INSSJP-PAMI.

Confidentiality {27}

Personal information needed to track the prescriptive behavior of physicians during the trial period is managed only by the principal investigator (FT) and converted to a numerical ID for all the remaining analysis.

Plans for collection, laboratory evaluation and storage of biological specimens for genetic or molecular analysis in this trial/future use {33}

Not applicable.

Statistical methods

Statistical methods for primary and secondary outcomes {20a}

The primary outcome will be analyzed using a mixed effects repeated measures ANOVA model. Comparisons between groups will be made using one-way ANOVA, while a multiple linear regression analysis will be implemented to evaluate the predictive value of the group condition controlling for baseline prescription levels. Finally, the Pearson chi-square test will be employed to analyze categorial variables.

To estimate the annual monetary savings attributable to our intervention at the national level, we will make the following calculations: first, we will estimate the number of items that would have been prescribed in the absence of the treatment. This number corresponds to the multiplication of the treatment effect times the number of items prescribed by an average physician in the control group during one year. We then multiply this quantity by the direct average price of each item (in U$S), according to the registers of the INSSJP-PAMI. This gives an estimated benefit per-physician/year. Should this intervention be implemented for all eligible physicians in the country during a year, *the total estimated benefit* is projected as the product between the benefit per-physician/year and the total number of doctors included in the study in both groups. As opposed to other similar interventions (such as Hallsworth et al., 2016) we will not incur in any substantial per-physician cost, as we will implement our treatment via e-mail instead of post mail. The main cost would thus be related to the analysis of the data to retrieve the top 25% of high-prescribers and the design of the letter. Being conservative, we budget as *total estimated cost* two full-time weeks of work (as we sent two communications) of a public servant for these tasks according to national statistics. Finally, the total estimated cost-benefit of the intervention is the *total estimated benefit* minus the *total estimated cost*.

Interim analyses {21b}

Three months after the first e-mail was sended we will carry an interim analysis to identify the participants of the treatment arm that reached a threshold of 10% reduction of the prescriptions. Those who reach the threshold will receive the “acknowledgement” version of the second e-mail,

meanwhile the remaining participants will receive the "encouragement" version.

Methods for additional analyses (e.g. subgroup analyses) {20b}

We will perform a sub-sample analysis of the physicians that opened the e-mails, which is a measure of the potential effect that the procedure could have on those actually reached by the intervention.

Methods in analysis to handle protocol non-adherence and any statistical methods to handle missing data {20c}

Because all data is obtained from the prescriptions' database from the INSSJP-PAMI we do not expect serious problems related with protocol non-adherence or missing data. If, despite this, there were problems related to missing data, they will be solved with multiple imputation methods.

Plans to give access to the full protocol, participant level-data and statistical code {31c}

Full protocol will be submitted to publication together with the manuscript reporting the results of the study as supplementary material.

INSSJP-PAMI regulations and the agreement signed with the institution does not allow researchers to share or make public the participant-level data set of the study.

Oversight and monitoring

Composition of the coordinating centre and trial steering committee {5d}

FT as principal investigator has the role of study co-ordinator and data manager. The Trial Management Group will be responsible for the day-to-day running and management of the trial and is integrated by FT, JB, FTR and RM.

Composition of the data monitoring committee, its role and reporting structure {21a}

DMC is not needed because there are no expected adverse events or harms and because of the short duration of the intervention.

Adverse event reporting and harms {22}

There are no expected adverse events or harms.

Frequency and plans for auditing trial conduct {23}

Because of the short duration of the intervention period and the simplicity of the procedures, there is no plan for auditing trial conduct.

Plans for communicating important protocol amendments to relevant parties (e.g. trial participants, ethical committees) {25}

Any changes will be communicated and submitted to consensus with the authorities of the INSSJP-PAMI.

Dissemination plans {31a}

Results of the study will be published at peer reviewed journals and presented in relevant academic events. Publication need the approval of INSSJP-PAMI authorities.

Declarations

Authors' contributions {31b}

FT, FLB, NA, JB, and FTR conceived the study. All authors contributed to the study design. FT, FLB, and NA developed the trial protocol.

Funding {4}

The trial is partially funded by the Interamerican Development Bank.

Availability of data and materials {29}

Due to legal restrictions emanating from the INSSJP-PAMI regulations, supporting data cannot be made openly available. All other relevant data will be included in the final article and/or its supplementary information files.

Ethics approval and consent to participate {24}

The study was approved by the Ethics Committee of Fundación INECO and the Department of

Legal Affairs of the INSSJP-PAMI.

Consent for publication {32}

Not applicable.

Competing interests {28}

We declare no competing interests.

References

Farooq MU, Min J, Goshgarian C, Gorelick PB. Pharmacotherapy for vascular cognitive impairment. CNS drugs. 2017; 31(9): 759-776.

Gorelick PB, Scuteri A, Black SE, et al. Vascular contributions to cognitive impairment and dementia: A statement for healthcare professionals from the American Heart Association/American Stroke Association. Stroke. 2011; 42: 2672-713.

Hallsworth M, Chadborn T, Sallis A, et al. Provision of social norm feedback to high prescribers of antibiotics in general practice: a pragmatic national randomised controlled trial. Lancet. 2016; 387(10029):1743-1752.

John P, Sanders M, Wang J. The use of descriptive norms in public administration: a panacea for improving citizen behaviours? 2014; Available at SSRN 2514536.

Liao JM, Navathe AS. Nudging physicians to reduce quetiapine prescribing using Medicare letters: following the letters of the law? JAMA Psychiatry. 2018; 75(10): 989-990.

McKeith IG, Boeve BF, Dickson DW, et al. Diagnosis and management of dementia with Lewy bodies: Fourth consensus report of the DLB Consortium. Neurology. 2017; 89(1): 88-100. doi: 10.1212/WNL.0000000000004058.

Meeker D, Linder JA, Fox CR, et al. Effect of behavioral interventions on inappropriate antibiotic prescribing among primary care practices: a randomized clinical trial. JAMA. 2016; 315(6):562-570. doi:10.1001/jama.2016.0275.

National Institute for Health and Care Excellence (NICE). Dementia: assessment, management and

support for people living with dementia and their carers (NG7). NICE guideline. 2018; Published: 20 June 2018. nice.org.uk/guidance/ng97

Peters R, Booth A, Peters J. A systematic review of calcium channel blocker use and cognitive decline/dementia in the elderly. Journal of Hypertension. 2014; 32(10): 1945-1958.

Petersen RC, Lopez O, Armstrong MJ et al. Practice guideline update summary: Mild cognitive impairment: Report of the Guideline Development, Dissemination, and Implementation Subcommittee of the American Academy of Neurology. Neurology. 2018; 90(3):126-135. doi: 10.1212/WNL.0000000000004826.

Prince M, Wimo A, Guerchet M, et al. World Alzheimer Report 2015: The Global Impact of Dementia. [online] Alzheimer's Disease International. Available at: https://www.alz.co.uk/research/WorldAlzheimer Report 2015.pdf. Last accessed 5/31/2016.

Rojas G, Demey I, Arizaga RL. Drugs used for cognitive impairment. Analysis of 1.5 million prescriptions in Argentina, Medicina. 2013; 73(3): 213-23.

Rojas G, Serrano C, Dillon C, et al. Use and abuse of drugs in cognitive impairment patients. VERTEX Rev. Arg. de Psiquiatría. 2010; 21: 18 – 23.

Sacarny A, Barnett ML, Le J, Tetkoski F, Yokum D, Agrawal S. Effect of peer comparison letters for high-volume primary care prescribers of quetiapine in older and disabled adults: a randomized clinical trial. JAMA Psychiatry. 2018; 75(10): 1003-101.

Sanders M, Snijders V, Hallsworth M. Behavioural science and policy: where are we now and where are we going? Behavioural Public Policy. 2018; 2(2): 144-167.

Schmidt R, Hofer E, Bouwman FH, et al. EFNS-ENS/EAN Guideline on concomitant use of cholinesterase inhibitors and memantine in moderate to severe Alzheimer's disease. Eur J Neurol. 2015; 22(6): 889-98. doi: 10.1111/ene.12707.
