# Supplementary material for: Effect of a Social Norm Email Feedback Program on the Unnecessary Prescription of Nimodipine in Ambulatory Care of Older Adults: A Randomized Clinical Trial
Source: JAMA Netw Open. 2020 Dec 11;3(12):e2027082. doi: 10.1001/jamanetworkopen.2020.27082 (PMC7733153; doi:10.1001/jamanetworkopen.2020.27082)
Supplement: Supplement 2. — eAppendix 1. First Active Letter eAppendix 2. First Control Letter eAppendix 3. Second Active Letter: Acknowledgement Version eAppendix 4. Second Active Letter: Encouragement Version eAppendix 5. Second Control Letter eTable 1. Rate of Opening the First Email eTable 2. Rate of Opening the Second Email eTable 3. Global Rate of Opening the Emails eTable 4. Expenditures of Nimodipine Prescriptions eAppendix 6. Additional Statistics [file jamanetwopen-e2027082-s002.pdf]

Supplemental Online Content

Torrente F, Bustin J, Triskier F, et al. Effect of a social norm email feedback program on the unnecessary prescription of nimodipine in ambulatory care of older adults: a randomized clinical trial. *JAMA Netw Open*. 2020;3(12):e2027082. doi:10.1001/jamanetworkopen.2020.27082

eAppendix 1. First Active Letter
eAppendix 2. First Control Letter
eAppendix 3. Second Active Letter: Acknowledgement Version
eAppendix 4. Second Active Letter: Encouragement Version
eAppendix 5. Second Control Letter
eTable 1. Rate of Opening the First Email
eTable 2. Rate of Opening the Second Email
eTable 3. Global Rate of Opening the Emails
eTable 4. Expenditures of Nimodipine Prescriptions
eAppendix 6. Additional Statistics

This supplemental material has been provided by the authors to give readers additional information about their work.

© 2020 Torrente F et al. *JAMA Network Open.*

eAppendix 1. First Active Letter

PAMI INSSJP NEWS

Nimodipine Prescription

Cognitive Impairment and dementias

Dear Doctor:

At PAMI we are promoting the appropriate prescription for older adults and we want to help you in this task.

An analysis conducted on the prescription of nimodipine shows that **you prescribe more nimodipine than the average of PAMI doctors**

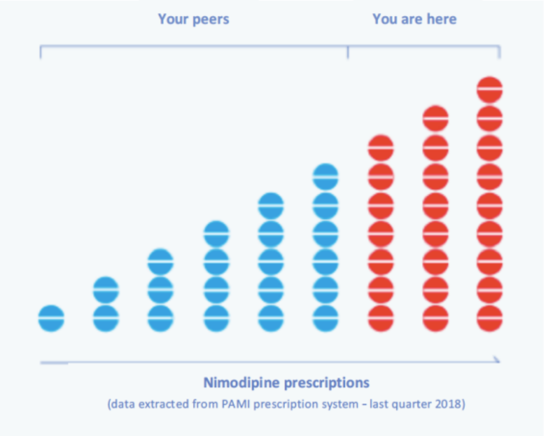

Current medical evidence shows that NIMODIPINE:

- **It is not effective** in preventing or treating cognitive decline and dementias (Farooq et al., 2017; Peters et al., 2014, Gorelick et al., 2011)
- It has a single evidence-based indication: ischemic disorders in patients with subarachnoid hemorrhage resultant from congenital aneurysms. Therefore, its use should be infrequent in the outpatient setting.

For this reason, we invite you to revise the indication for NIMODIPINE in your next consultations and consider whether it can be discontinued in patients who currently receive it and do not meet the indication criteria.

For the management of patients with cognitive impairment, you can consult the Guide for GPs on Mild Cognitive Impairment and Dementias on the PAMI GPs website or the NICE Guidelines on Dementias.

Likewise, we recommend that you consult the Medication Prescription Guide, where you will find information about the different drugs that may carry excessive risks or be inappropriate for those over 65 years of age.

We appreciate your commitment to provide our patients the care they need.

Dr. Ricardo Mastai
Medical director
INSSJP - PAMI

www.pami.org.ar /pami.org.ar PAMI_org_ar

© 2020 Torrente F et al. *JAMA Network Open.*

eAppendix 2. First Control Letter

PAMI INSSJP

NEWS

Drugs Prescription

Rates of use and polypharmacy

Dear Family Doctor:

In order to take care of the health of our members and promote prescription based on the best available evidence, we bring you the following information:

- Prescribing medications is a medical act that can generate benefits, but also serious risks to people.
- Adverse drug reactions are highly prevalent in people over 65 years of age and generate between 20% and 30% of hospital intakes in older patients.
- Approximately 1 in 5 prescriptions for older people in primary care is inappropriate and frequently leads to polypharmacy and consequent morbidity and mortality.

Therefore, we advise NOT to start a medication unless it is strictly necessary and SUSPEND any medication that is not essential. Likewise, it is important to bear in mind the need to adapt the drugs to the physiological changes associated with aging and the close monitoring of those that require it.

Suspension of medications should be considered in the following circumstances:

- Polymedicated patients.
- When drugs produce adverse effects.
- When drugs have not shown efficacy in clinical trials.
- When drugs are not effective in the patient.
- When life expectancy is short: terminally ill or patients with advanced dementia.
- When medications are no longer necessary or appropriate to the patient's clinical situation.
- When duplications, relevant interactions, prescription errors, inadequate medication are detected.

For more information on the different drugs that may carry excessive risks or be inappropriate in older people, you can consult the Medication Prescription Guide on the PAMI Portal for Primary Care Physicians.

We appreciate your commitment to provide our members with the care they need.

Dr. Ricardo Mastai
Medical director
INSSJP - PAMI

www.pami.org.ar /pami.org.ar PAMI_org_ar

© 2020 Torrente F et al. *JAMA Network Open.*

eAppendix 3. Second Active Letter: Acknowledgement Version

PAMI INSSJP

NEWS

Nimodipine Prescription

Cognitive Impairment and dementias

In May we contacted you to provide information about nimodipine prescription.

Since that time we have noticed a reduction in your prescriptions of nimodipine in our electronic prescription registry.

We want to congratulate and thank you for your commitment to carry out a rational and evidence-based practice for the benefit of our patients.

Best regards,

Dr. Ricardo Mastai
Medical director
INSSJP - PAMI

www.pami.org.ar /pami.org.ar PAMI_org_ar

© 2020 Torrente F et al. *JAMA Network Open.*

eAppendix 4. Second Active Letter: Encouragement Version

PAMI INSSJP

NEWS

Nimodipine Prescription

Cognitive Impairment and dementias

Dear Doctor:

As we informed you in May, **you prescribe more nimodipine than the average of PAMI doctors**

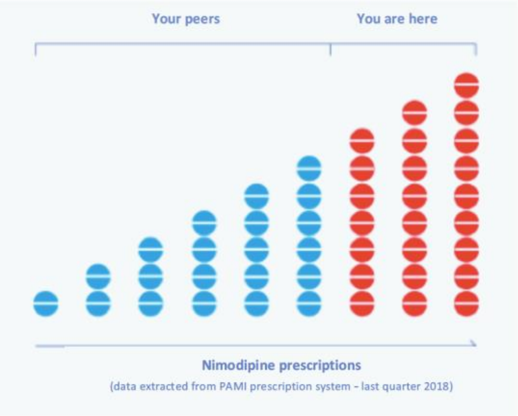

Current medical evidence shows that NIMODIPINE:

- **It is not effective** in preventing or treating cognitive decline and dementias (Farooq et al., 2017; Peters et al., 2014, Gorelick et al., 2011)
- It has a single evidence-based indication: ischemic disorders in patients with subarachnoid hemorrhage resultant from congenital aneurysms. Therefore, its use should be infrequent in the outpatient setting.

Many of your colleagues who received the notification significantly reduced prescriptions. For this reason, we invite you again to revise the indication for NIMODIPINE in your next consultations and consider whether it can be discontinued in patients who currently receive it and do not meet the indication criteria.

For the management of patients with cognitive impairment, you can consult the Guide for GPs on Mild Cognitive Impairment and Dementias on the PAMI GPs website or the NICE Guidelines on Dementias.

Likewise, we recommend that you consult the Medication Prescription Guide, where you will find information about the different drugs that may carry excessive risks or be inappropriate for those over 65 years of age.

We appreciate your commitment to provide our patients the care they need.

Dr. Ricardo Mastai
Medical director
INSSJP - PAMI

www.pami.org.ar /pami.org.ar PAMI_org_ar

eAppendix 5. Second Control Letter

PAMI INSSJP

NEWS

Drugs Prescription

The challenge of benzodiazepines

Dear Doctor:

In order to take care of the health of our members and promote evidence-based prescription, we bring you the following information:

- Benzodiazepines are the most prescribed psychotropic drugs in the PAMI, representing 67% of the psychotropic drug prescriptions.

- In 2016, clonazepam and alprazolam constituted 54% of the total of the prescribed psychotropic drugs.

- There is enough evidence that shows that the prolonged use of benzodiazepines produces alterations in attention and memory, and increases the risk of traffic accidents, cognitive deterioration, ataxia, falls, hip fracture, hypoventilation, increased mortality, tolerance for their sedative effects and withdrawal syndromes.

- According to the STOPP-START criteria proposed by the British Geriatrics Society, benzodiazepines should not be used for a treatment period longer than four weeks.

- In 2015, the United States Agency for Research and Quality of Health Care reported little or no evidence of long-term efficacy in benzodiazepine treatments for the management of insomnia disorder.

- There is no medication for primary or chronic insomnia in older people that has been shown to be safe and effective.

To avoid inappropriate prescription and polypharmacy, we advise NOT to start a medication unless it is unavoidable and to SUSPEND OR GRADUALLY DISCONTINUE any medication that is not essential. Likewise, it is important to bear in mind the need to adapt the drugs to the physiological changes associated with aging and the close monitoring of those that require it.

For more information on the different drugs that may carry excessive risks or be inappropriate in people over 65, you can consult the Guide to Prescribing Medicines.

We appreciate your commitment to provide our members with the care they need.

Dr. Ricardo Mastai
Medical director
INSSJP - PAMI

www.pami.org.ar /pami.org.ar PAMI_org_ar

© 2020 Torrente F et al. *JAMA Network Open.*

eTable 1. Rate of Opening the First Email

	Type of letter		
	**Active (treatment)**	**Control**	**Total**
	**n (%)**	**n (%)**	**n (%)**
Open	335 (37.0)	349 (38.6)	684 (37.8)
Not open	571 (63.0)	556 (61.4)	1127 (62.2)
Total	906 (100.0)	905 (100.0)	1811 (100.0)

eTable 2. Rate of Opening the Second Email

	Type of letter			
	**Control**	**Acknowledgement**	**Encouragement**	**Total**
	**n (%)**	**n (%)**	**n (%)**	**%**
Open	307 (33.9)	142 (79.8)	211 (29.0)	660 (36.49)
Not open	598 (66.1)	36 (20.2)	517 (71.0)	1151(63.69)
Total	905 (100.0)	178 (100.0)	728 (100.0)	1811(100.0)

eTable 3. Global Rate of Opening the Emails

**Opened**	**Control n (%)**	**Treatment n (%)**
1st and 2nd e-mail	230 (25.4)	261 (28.8)
1st e-mail only	119 (13.1)	74 (8.2)
2nd e-mail only	77 (8.5)	92 (10.2)
Either 1st or 2nd	426 (47.1)	427 (47.1)
None	479 (52.9)	479 (52.9)

eTable 4. Expenditures of Nimodipine Prescriptions

	**Expenditure, Argentinean Pesos**		
	Control	Treatment	Treatment - Control
Baseline period 1[a]	61117887	59027125	- 2090762
Baseline period 2[b]	60494466	59291407	- 1203059
Intervention period[c]	59290929	55035885	- 4255044

[a] May–October 2018; [b] November 2018–April 2019; [c] May–October 2019.

© 2020 Torrente F et al. *JAMA Network Open.*

eAppendix 6. **Additional Statistics**

1.1. *Extended subgroup analysis of outcomes*

We performed two subgroup analysis. Partial sample 1 included the participants that opened the first e-mail (treatment group, n=335; control group, n=349), meanwhile partial sample 2 included those who opened the first or the second e-mail (treatment group, n=427; control group, n=426). eTable 9 shows total and average number of prescriptions for the two subsamples. Treated physicians from partial sample 1 prescribed 13.77% less nimodipine units than controls (28701 versus 33284; mean difference per prescriber, -9,69; 95% CI, -18.50 to -0.89; p = .03), and those included in partial sample 2 prescribed 11.11% less (36116 vs 40625; mean difference per prescriber, -10.78; 95% CI, -18.53 to -3.03; p = .006). Regression analysis for partial sample 2 revealed a significant association of the group condition with nimodipine prescriptions per 1,000 total prescriptions during the intervention period when controlling for baseline prescriptions [B = -0.671; 95% CI, -0.900 to -0.442, $p < .001$, R^2 =.816]

	**Total no. of prescriptions**			**Per prescriber prescriptions**			
	**Control**	**Treatment**	**Difference**	**Control**	**Treatment**	**Difference**	***P***
	No.	No.	No. (%)	Mean (95% CI)	Mean (95% CI)	Mean (95% CI)	
Total sample[1]	89588	84489	-5099 (-5.69)	98.99 (95 – 102.98)	93.25 (89.27 – 97.24)	-5.73 (-11.38 – -0.10)	.046
Partial Sample 1[2]	33284	28701	-4583 (-13.77)	95.37 (89.21 – 101.53)	85.67 (79.38 – 91.96)	-9.69 (-18.50 – -0.89)	.03
Partial Sample 2[3]	40625	36116	-4509 (-11.10)	95.36 (89.88 – 100.85)	84.58 (79.10 – 90.06)	-10.78 (-18.53 – -3.03)	.006

1.2. *Factors related with opening rate of the e-mails*

© 2020 Torrente F et al. *JAMA Network Open.*

We performed several additional analyses comparing the characteristics of the physicians that opened the e-mails with those who did not open the e-mails.

1.2.1 Age

We found significant between groups differences regarding age when comparing the physicians that opened with those who did not open the e-mails (see below).

Age			
	**Mean**	**SD**	**N**
Opened	55.382	10.684	841
Not opened	58.044	10.371	951

ANOVA - Age					
**Cases**	**Sum of Squares**	**df**	**Mean Square**	**F**	**p**
Opening	3163.805	1	3163.805	28.590	< .001
Residuals	198080.623	1790	110.660		
Note. Type III Sum of Squares					

1.2.2 Years of practice

We found significant between groups differences regarding years of practice at the institute when comparing the physicians that opened with those who did not open the e-mails (see below).

© 2020 Torrente F et al. *JAMA Network Open.*

Years of Practice			
	**Mean**	**SD**	**N**
Opened	7.960	3.199	853
Not opened	8.947	2.889	958

ANOVA – Years of Practice					
**Cases**	**Sum of Squares**	**df**	**Mean Square**	**F**	**p**
Opening	439.237	1	439.237	47.566	< .001
Residuals	16704.930	1809	9.234		
Note. Type III Sum of Squares					

1.2.3 Gender

Rate of opening was associated with gender. Female physicians opened the e-mails more frequently than males:

Contingency Tables			
	**Gender**		
	**Female**	**Male**	**Total**
Not opened	327	631	958
Opened	358	495	853
Total	685	1126	1811

Chi-Squared Tests			
	**Value**	**df**	**p**
X^2	11.781	1	< .001
N	1811		

© 2020 Torrente F et al. *JAMA Network Open.*

Nominal	
	**Value**
Phi-coefficient	0.081
Cramer's V	0.081

1.2.4 Region

We grouped physician in six geographic regions according to their location of practice (Northwest, Northeast, Pampa region, Cuyo region, Metropolitan Region of Buenos Aires and Patagonia), Rate of opening was no associated with the region of practice of the physicians.

	**REGION**						
	**NOA**	**NEA**	**PAMPA**	**CUYO**	**RMBA**	**PAT**	**Total**
Not opened	103	49	369	59	376	2	958
Opened	69	37	319	43	381	4	853
Total	172	86	688	102	757	6	1811

NOA= Northwest, NEA=Northeast, RMBA= Metropolitan Region of Buenos Aires, PAT=Patagonia.

Chi-Squared Tests			
	**Value**	**df**	**p**
X^2	9.182	5	0.102
N	1811		

© 2020 Torrente F et al. *JAMA Network Open.*
