# Supplementary material for: Effect of a Social Norm Email Feedback Program on the Unnecessary Prescription of Nimodipine in Ambulatory Care of Older Adults: A Randomized Clinical Trial
Source: JAMA Netw Open. 2020 Dec 11;3(12):e2027082. doi: 10.1001/jamanetworkopen.2020.27082 (PMC7733153; doi:10.1001/jamanetworkopen.2020.27082)
Supplement: Supplement 3. — Data Sharing Statement [file jamanetwopen-e2027082-s003.pdf]

Data Sharing Statement

Torrente. Effect of a Social Norm Email Feedback Program on the Unnecessary Prescription of Nimodipine in Ambulatory Care of Older Adults. *JAMA Netw Open*. Published December 11, 2020. doi:10.1001/jamanetworkopen.2020.27082

Data
Data available: No

Additional Information
Explanation for why data not available: Regulations and the agreement signed with the institution where the study was carried (INSSJP-PAMI, Argentina) do not allow researchers to share or make public the participant-level data set of the study.
